# Mitogenomic Relationships and Demographic History of the Daurian Ground Squirrel (*Spermophilus dauricus*) in Response to Human Activity

**DOI:** 10.1002/ece3.72605

**Published:** 2025-11-28

**Authors:** Xi Chen, Zhenshan Liu, Ming Yang, Yu Zhou

**Affiliations:** ^1^ College of Life Sciences Shenyang Normal University Shenyang China

**Keywords:** Illumina sequencing, mitochondrial genome, population genetics, species distribution modeling

## Abstract

Species are the constituent units of ecosystems, and even species that have severe conflicts with humans play irreplaceable and important roles in maintaining ecosystem stability. *Spermophilus dauricus* has long been considered a harmful animal because of its ability to damage grasslands and agriculture, as well as its status as a primary host of *Yersinia pestis* . Consequently, it has been subject to eradication areas in human activity areas, which may lead to a reduction in its genetic diversity. Genetic diversity is the core foundation for maintaining species continuity, yet this is precisely the aspect lacking in research on *S. dauricus* . In this study, we performed mitochondrial genome sequencing of 73 individuals sampled across an extensive geographic region of *S. dauricus* and conducted population genetic and species distribution model analyses. The results revealed that *S. dauricus* is primarily distributed in three fragmented yet connected regions: the Northeast China Plain distribution area, the Hulunbuir Plateau distribution area, and the Bashang Plateau distribution area. Populations across these three present regions exhibit genetic differences but do not display subspecific relationships. The populations in the Hulunbuir Plateau and Bashang Plateau likely originated from the population in the Northeast Plain. Furthermore, all three populations of *S. dauricus* have experienced a continuous population decline in the past few thousand years, which we hypothesize may be related to the development of human rice cultivation and nomadism over the past few thousand years. This study highlights the role of human production activities in the decline of the effective population of rodents.

## Graphical abstract

An analysis of population genetics on the basis of the mitochondrial genomes of widely sampled *Spermophilus dauricus* , integrated with the results of species distribution models, reveals the following: There are differences in population differentiation among the three *Spermophilus dauricus* populations in the Northeast Plain, the Hulunbuir Plateau, and the Bashang Plateau, but these differences do not constitute a subspecies relationship. The population of *S. dauricus* has experienced a significant decline over the past few thousand years, which may be caused by the development of human grazing and rice cultivation.

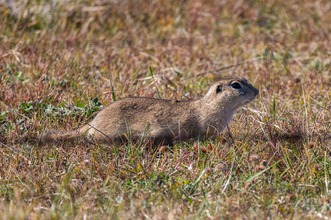

## 1 Introduction

**FIGURE 1:**
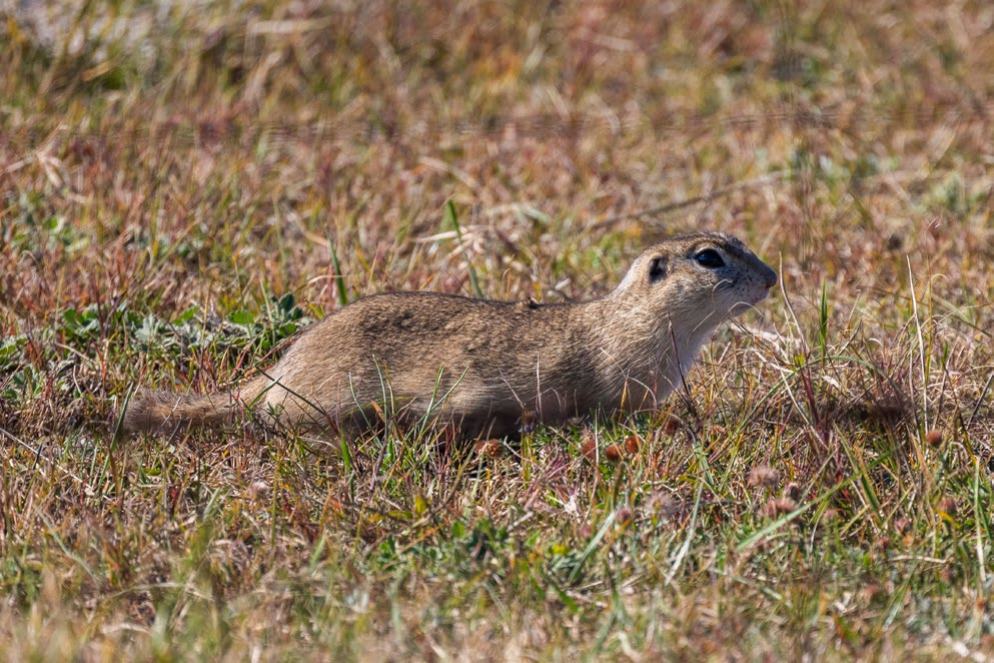
An individual of *Spermophilus dauricus* Brandt, 1843 from Hexigten Banner, Chifeng, Inner Mongolia, China. Photograph by Xiaotian Xing (Email: 864932777@qq.com) and published with his permission.

On the basis of revisions in phylogenetic biology (Helgen et al. 2009; Simonov et al. 2024), there are currently 18 known species worldwide in the genus *Spermophilus*. *Spermophilus dauricus* Brandt 1843 (Figure 1) is one of the most widespread *Spermophilus* species, distributed at the northeasternmost end of the entire distribution range of the genus *Spermophilus*, primarily in northeastern China as well as in the border regions of China, Mongolia, and Russia (Wang 2003; Wei 2022). With respect to the internal subspecific composition of *S. dauricus* , at least four subspecies have been described: *S. dauricus dauricus*, the type subspecies of the Daurian ground squirrel, which is distributed in the vicinity of Torey Lakes in Transbaikalia; *S. dauricus mongolicus*, which is distributed in the regions of Hebei, Henan, and Shandong Provinces; *S. dauricus ramosus*, which is distributed in the Northeast Plain and eastern Inner Mongolia; and *S. dauricus obscurus*, which is distributed in northwestern Gansu and northern Xinjiang (Wang 2003; Wei 2022). More studies indicate that the habitat of *S. dauricus* is restricted to areas from northeastern China to Hebei Province (Jiang 2021; Simonov et al. 2024). Moreover, the distribution area of the Gansu subspecies, i.e., the Altai Mountains region, is currently recognized as the range of *Spermophilus alashanicus* (Simonov et al. 2024; Wei 2022). Therefore, the main question regarding the subspecific differentiation of this species is whether the three geographical populations in the Hulunbuir Plateau, the Northeast Plain, and the northern parts of Hebei and Shanxi Provinces constitute distinct subspecies (Kapustina et al. 2018; Wei 2022).

Burrowing rodents are typical ecosystem engineers. They alter soil structure through burrowing, improving soil aeration and water permeability. Meanwhile, the microhabitat provided by burrows can also serve as shelters for other species, indirectly promoting the maintenance of community species diversity (Prugh and Brashares 2012; Reichman and Seabloom 2002). In addition, the burrowing activities of burrowing rodents play a key role in driving the material cycle and energy flow of ecosystems. For example, soil tillage during burrowing can accelerate the decomposition of organic matter and nutrient cycling; at the same time, the differences in surface microtopography formed by burrows can create diverse microenvironments for plant seed germination (Van Nimwegen et al. 2008; Zhang et al. 2003). *S. dauricus* primarily inhabits arid and semiarid grasslands, semidesert areas, and adjacent agro‐pastoral ecotones. As an important component of grassland ecosystems, it plays multiple roles in ecological dynamics. First, as a primary consumer, the omnivorous *S. dauricus* feeds on tender plant parts and seeds, making it a pest in agriculture and animal husbandry (Gür and Gür 2010; Wang, Ji, et al. 2015). However, insects and other small invertebrates also constitute a major part of its diet, which helps prevent the outbreak of grassland insect infestations (Streubel and Fitzgerald 1978; Wang, Ji, et al. 2015). On the other hand, rodents are among the most significant prey groups in ecosystems, and their population size and dynamics directly affect the survival and reproduction of carnivores and birds of prey, playing crucial roles in maintaining the integrity of food chains (Baatargal and Suuri 2021). In addition to damaging grasslands and agricultural crops, *S. dauricus* is also a primary natural host of *Yersinia pestis* , and has long been regarded by humans as a major harmful animal (Zhang et al. 2007).

Even species that conflict with humans can provide important ecosystem functions. As a species that significantly conflicts with humans, understanding the population genetic relationships, genetic diversity, and population historical dynamics of *S. dauricus* is highly important, both for its control and for avoiding the loss of diversity caused by excessive eradication. In this study, we conducted extensive sampling of *S. dauricus* and, by combining mitochondrial genome sequencing with species distribution modeling, revealed the current status of its population genetics as well as the causes underlying this population's genetic status.

## 2 Materials and Methods

### 2.1 Sampling, Laboratory Procedures and Sequencing

**FIGURE 2:**
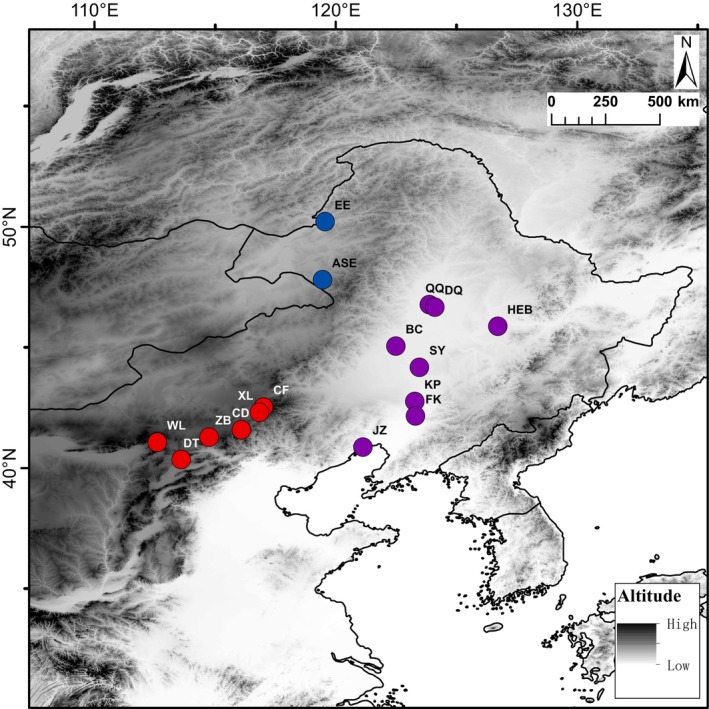
Distribution of the sampling sites for the three populations of *S. dauricus* . The site codes are also present in Table S1.

Seventy‐three samples from 16 locations were collected for our study (Table S1 and Figure 2). The animals were treated in accordance with the guidelines of the Ethics Committee of the College of Life Sciences, Shenyang Normal University (Ethical Approval Number SYNU‐2409‐008), which approved this study. For each sample, venous blood samples (~50 μL) were collected from the jugular vein of *S. dauricus* via disposable venous blood collection tubes. The blood samples were immediately preserved in a car refrigerator at −20°C. Total genomic DNA was extracted from ethanol‐preserved tissues via the TIANamp Genomic DNA Kit (Beijing, China). Each mitochondrial genome of the 72 *S. dauricus* strains was amplified via two long‐PCRs (for information on the custom‐designed long‐PCR primers, see Table S2). TaKaRa LA Taq (Takara Inc., Dalian, China) was used for long‐range PCR. Thermal cycling began with a denaturation period of 5 min at 95°C that was followed by 30 cycles of 94°C for 1 min, annealing at 55°C for 1 min, and 72°C for 10 min, with a final extension at 72°C for 20 min.

The mitogenome DNA pool of each sample was prepared according to the parallel tagged amplicon sequencing method described by Zhou et al. (2024). A total of 10 μg of the final pooled DNA was sent to Sangon Biotech (Shanghai, China) for Illumina HiSeq sequencing. The sequencing library was sequenced on an Illumina HiSeq 2500 sequencer. Approximately 15 GB of paired‐end 150‐bp clean reads (filtering low‐quality data) were obtained.

### 2.2 Read Sorting and Assembly

Paired‐end reads that contained a complete 10‐base species‐specific tag in at least one direction were retained for later analysis. First, we use the fsplit (https://github.com/yodeng/fsplit) script to extract individual‐specific sequences from the forward and reverse read FASTQ files, separately, on the basis of individual‐specific tags. The specific parameters include “fsplit split ‐i ‐b ‐o ‐d”. Then, use the seqkit script (Shen et al. 2024) to extract sequence IDs from each of the two filtered read FASTQ files of each individual, followed by merging and deduplication of these IDs. The specific parameters include “seqkit fx2tab ‐n >”. Finally, use the filterbyname script of BBMap (Bushnell 2014) to extract paired‐end FASTQ sequences on the basis of the individual ID files from the original paired‐end FASTQ files.

Individual‐specific tags and PCR primers were removed via Cutadapt v1.8.2 (Martin 2011). The mitochondrial genome of each individual of *S. dauricus* was assembled via GetOrganelle (Jin et al. 2020) following the recommended protocols for animal mitochondrial genome assembly. The raw read data for each individual is downloadable from the NCBI Sequence Read Archive (SRA) repository (accession SRR35199706—SRR35199778).

### 2.3 Genetic Relationship Analyses

The complete mitogenomes were annotated via MITOS (Bernt et al. 2013). Nucleotide sequences were aligned using MAFFT v7.520 (Nakamura et al. 2018) with default parameters. The alignment results showed a large number of insertions and deletions (InDels) in the D‐loop region; therefore, this region was excluded from subsequent genetic analyses. Nucleotide diversity, pairwise *F*st, and molecular variance (AMOVA) (Excoffier et al. 1992) were calculated using Arlequin v3.5 (Excoffier and Lischer 2010).

NETWORK v10 (Bandelt et al. 1999) was employed to construct a median‐joining network (MJN) for the mitochondrial genomes. After generating the initial MJN, the Maximum Parsimony (MP) option was chosen to remove excessive links and median vectors (Polzin and Daneshmand 2003). The genetic distance matrix under the K80 model and principal coordinate analysis (PCoA) were performed via the R package ape (Paradis et al. 2019).

### 2.4 Historical Demography

The demographic history of each population and all *S. dauricus* samples was determined by means of neutrality tests and mismatch distributions in Arlequin (Excoffier and Lischer 2010). Fu's *F*s test (Fu 1997) and Tajima's *D* test (Tajima 1989) were applied to test whether the populations evolved under neutrality. Mismatch distributions (Harpending 1994) were constructed using the sudden expansion model of Schneider and Excoffier (1999) with 10,000 bootstrap replicates, and the validity of the sudden expansion assumption was determined using the sum of squares differences (*SSDs*) and Harpending's raggedness index (*Hri*) (Harpending 1994).

BEAST v2.7.6 (Bouckaert et al. 2014) was used to perform Bayesian skyline plot (BSP) analysis (Drummond et al. 2005) to estimate the change in effective population size over time and the time to the most recent common ancestor (tMRCA). The divergence time of 3.7 million years ago between the *S. dauricus* and the *S. alaschanicus*, as reported by Simonov et al. (2024), was used for BSP calibration. The mitogenome sequences of *S. alaschanicus* (NC_071768) were downloaded from GenBank (Zhao et al. 2024). The best substitution model was determined via the bModelTest package (Bouckaert and Drummond 2017) implemented in BEAST. Two independent analyses were performed using all the mitochondrial genome sequences available for each individual. MCMC was run with 10^9^ steps, with sampling every 10,000 steps. The results of each run were visualized via TRACER v1.7 (Rambaut et al. 2018) to ensure that stationarity and convergence had been reached and that the effective sample size (ESS) was greater than 200.

### 2.5 Species Distribution Modeling

Species distribution models (SDMs) were predicted for *S. dauricus* via the maximum entropy model implemented in Maxent v3.4.4 (Phillips et al. 2025). A total of 38 site data points with distances greater than 10 km between them were used for SDM analysis (Table S3). The site information is derived from our sampling sites and data from the iNaturalist website (https://www.inaturalist.org/), which has been filtered by the authors of this study on the basis of geographical distribution. Nineteen bioclimatic variables and elevation data (at a 30 arcsecond resolution) were downloaded from the WorldClim v2.1 database (Fick and Hijmans 2017). The normalized difference vegetation index (NDVI) in July 2024 was obtained from the NASA LPDAAC collection in the MODIS database (https://lpdaac.usgs.gov). The global land cover types in 2001 and 2010 (Wang, Zhao, et al. 2015) were downloaded from the iEarth DataHub (https://data‐starcloud.pcl.ac.cn/iearthdata).

The contribution percentage and permutation importance analysis of the environmental variables were assessed using Maxent. First, the environmental factors with a contribution percentage of “0” were removed, and a total of 22 environmental variables were obtained (Table S4). Second, to determine and exclude extremely correlated variables, ENMTools v1.3 was used to perform correlation analysis between the 22 variables. The correlation coefficient |*R*| < 0.8 is used as a cutoff to minimize the effects of multicollinearity and model overfitting (Elith et al. 2010). Finally, the variable with the highest contribution percentage was selected from those variables with |*R*| > 0.8 (Figure S1) and used for Maxent modeling to avoid overfitting. Different regularization multipliers (0.5, 1, 1.5, 2, 2.5, 3, 3.5, and 4) and feature classes were assessed in R via the “kuenm” package (Cobos et al. 2019) to obtain the best combination of runs in Maxent. We adopted the default settings of kuenm (Cobos et al. 2019), and the candidate model performance was evaluated on the basis of significance (partial receiver operating curve (ROC), 100 iterations, and 50% of the data for bootstraps), omission rate (*E* = 5%), and model complexity. The Akaike information criterion correction (AICc) is a standard for measuring the goodness of fit of a statistical model and can be used to weigh the complexity of the estimated model and the goodness of fit of the model data. This gives priority to the source with the smallest delta AICc Model (Akaike 1998). Therefore, when the omission rate was < 5% and the delta AICc was the smallest, the model parameters were optimal (Cobos et al. 2019), with the input of optimized parameters entered into Maxent (Figure S2). As a result, low multicollinearity (Figure S1) environment variables Annual Mean Temperature, Mean Diurnal Range, Isothermality, Temperature Seasonality, Max Temperature of the Warmest Month, Precipitation of the Wettest Month, Precipitation Seasonality, Precipitation of the Wettest Quarter, Precipitation of the Coldest Quarter, Land Cover, Elevation and Normalized Difference Vegetation Index, regularization multiplier 0.5, and feature class selected Linear and Quadratic features were used for final SDM analysis. The fitness indices, which represent the potentially suitable distribution range of *S. dauricus* , were obtained. Fitness indices (ranging from 0 to 1) > 0.5 indicate that the environment is very suitable for the species to inhabit. Four grades were used here: high suitability (> 0.5), medium suitability (0.3 ~ 0.5), low suitability (0.05 ~ 0.3) and no suitability (< 0.05) (Martín‐García et al. 2014; Zhou et al. 2023). Among the distribution points of *S. dauricus* used in the SDM analysis of this study (Table S3), over 60% fell within the threshold of high suitability areas, and more than 80% were within the medium and high suitability areas combined. Therefore, we consider the four‐grade habitat suitability classification in this study to be reasonable.

## 3 Results

### 3.1 Mitogenome Assembly, Genetic Diversity, and Differentiation

All samples in this study were successfully assembled into complete mitochondrial genome sequences, with a sequencing depth of > 50× for each. (GenBank accession numbers: PX251470–PX251542, Table S1). The aligned mitogenome dataset for *S. dauricus* (16,474 bp) yielded 48 haplotypes among 72 sequences. The nucleotide diversity of each population location and all the samples presented different values (Table 1). The lowest nucleotide diversity was 0.00169 in the NM population, the HB population had a relatively higher nucleotide diversity value of 0.00243, and the highest nucleotide diversity was 0.00630 in the DB population. The nucleotide diversity of all the samples was 0.00591 (Table 1).

**TABLE 1:** Summary statistics of the demographic analysis of *S. dauricus* .

Pop	*N*	*N*_*H*_	Tajima's *D* (*p*)	*Fu's Fs* (*p*)	*ND* (*π*)	*SSD* (*p*)	*Hri* (*p*)
NM	12	7	−1.491 (0.063)	3.732 (0.941)	0.00169	0.059 (0.000)	0.098 (0.000)
DB	34	23	−0.447 (0.371)	5.559 (0.976)	0.00630	0.015 (0.250)	0.015 (0.000)
HB	26	20	−1.729 (0.022)	1.433 (0.743)	0.00243	0.006 (0.550)	0.010 (0.450)
All	72	50	−0.922 (0.181)	2.534 (0.820)	0.00591	0.006 (0.500)	0.004 (0.000)

Abbreviations: *Hri* = Harpending's raggedness index, *N* = number of individuals, *ND* = nucleotide diversity, *NH* = number of haplotypes, *SDD* = difference in the sum of squares or mismatch distribution.

AMOVA revealed that within‐population diversity accounted for 60.75% of the overall variation (Table S5). The diversity among populations was relatively low, at 39.25%. Pairwise FST analysis revealed that the DB population and the HB population had the closest genetic relationships, with an *F*st value of 0.24 (Table S6). The genetic difference between the NM population and the HB population was the largest, with an *F*st value of 0.76. The *F*st value between the DB population and the NM population is 0.4.

### 3.2 Genetic Relationship of *S. dauricus*

**FIGURE 3:**
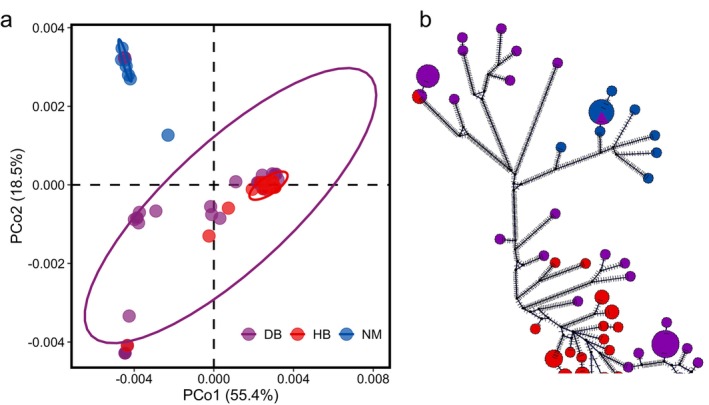
Principal coordinate analysis (PCoA) (a) and phylogenetic network analysis (b) of the three populations.

PCoA analysis revealed that the NM population and HB population each formed tight clusters, whereas the DB population was distributed (Figure 3a). The HB population clustered within the DB population. The NM population was significantly separated from the other populations. The percentage variance attributable to the two principal coordinate axes was 73.7% (axis 1: 54.9% and axis 2: 18.8%).

The phylogenetic network analysis more intuitively revealed the population genetic relationships of *S. dauricus* (Figure 3b). Within the NM population and HB population, individuals respectively maintain close genetic relationships with one another, and both populations are embedded in different positions within the DB population. The phylogenetic network analysis also revealed that the HB population had the closest genetic relationship with the Northeast population.

### 3.3 Historical Population Dynamics

The results of the neutrality test on *S. dauricus* showed that only Tajima's D value of the HB population was significantly negative (Table 1), indicating an excess of low‐frequency polymorphisms in this population relative to expectations, which suggests that the population has experienced expansion. In addition, the test results of *SSD* and *Hri* for the HB population were both nonsignificant, and the mismatch distribution curve showed a unimodal Poisson distribution (Figure S3). These findings indicate that its genetic structure has a high degree of fit with the population expansion model, supporting the conclusion that the HB population has experienced an expansion event. The nonsignificant Tajima's D and Fu's *F*s values of the NM, DB populations, and total population indicate that these populations may have been in a “stable state” for a long time.

**FIGURE 4:**
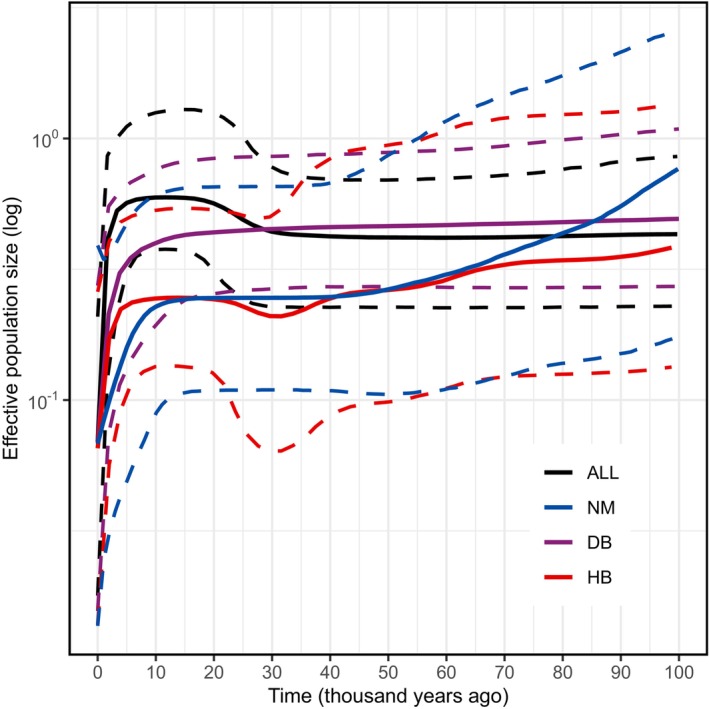
Bayesian skyline plot showing the historical demographic trends of each population and all samples of *S. dauricus* . The solid lines represent mean estimates, and the dashed lines represent 95% confidence intervals.

The BSP results suggested that the total population of *S. dauricus* has undergone a slight population expansion during 30,000 to 20,000 years ago (Figure 4). However, in the analysis of individual populations, only the HB population expanded during the same period, whereas the NM and DB populations maintained stable population dynamics during that time. Notably, both the analysis of the total population and the analysis of the historical population dynamics of each individual population revealed a drastic population contraction event in the recent period, with the DB and HB populations from approximately 4000 years ago to the present, whereas the NM population from approximately 10,000 years ago to the present.

### 3.4 Species Distribution Modeling of *S. dauricus*

The AUC is an efficient autonomous threshold index capable of evaluating the ability of a model to discriminate presence from absence. The results revealed that the 10‐repeated average values of AUC value were 0.958 (Figure S4), indicating that the constructed models were reliable and qualified. for the following predictions.

**FIGURE 5:**
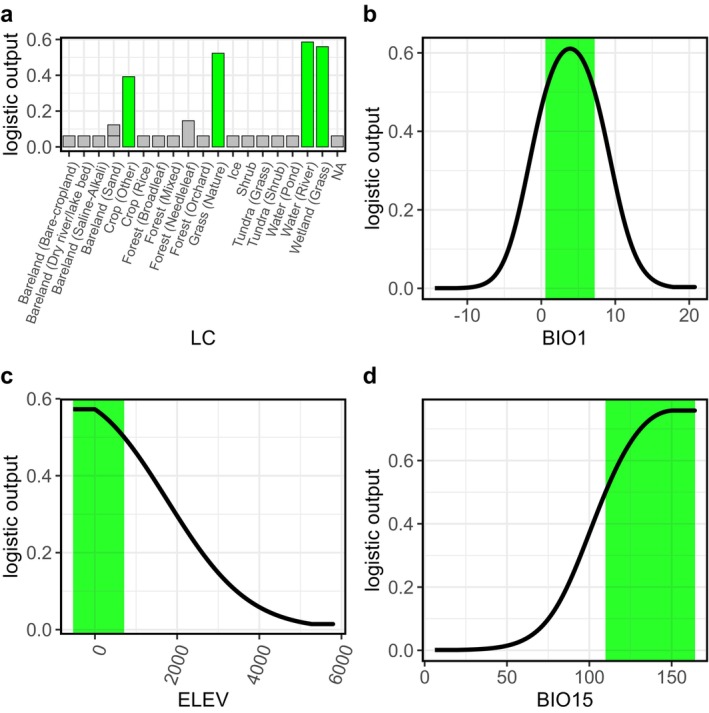
Species response curves of *S. dauricus* in relation to (a) LC, (b) BIO1, (c) ELEV, and (d) BIO15. The green shaded areas indicate logistic output values greater than 0.5. The meanings of the variable abbreviations are provided in Table S4.

The jackknife test of variable importance shows that the environmental variable with the highest gain when used in isolation is Land Cover, which therefore appears to have the most useful information by itself. The environmental variable that decreases the gain the most when it is omitted is Land Cover (36.7%), which therefore appears to have the most information that is not present in the other variables (Figure S5). The variable contribution analysis also revealed that the environmental factor Land Cover provided the highest contribution rates (Table S7). Next, the contribution rates in descending order were as follows: Annual Mean Temperature (13.5%), Elevation (11.1%), and Precipitation Seasonality (10.7%). On the basis of the species response curves acquired under current climatic conditions, *S. dauricus* preferred the grassland vegetation types, such as natural grass, wetland grass, and grasslands along the riverbanks, with the annual mean temperatures ranging from 0.56°C to 7.2°C, the elevation of −532 to 716 m, and the precipitation seasonality of 110–164 (Figure 5). In addition, in the species response curves for Land Cover, apart from the three factors with logistic output values greater than 50%, the logistic output values for the “other crop” factor (excluding Rice and Greenhouse) were also significantly higher than those for other vegetation types (Figure 5).

**FIGURE 6:**
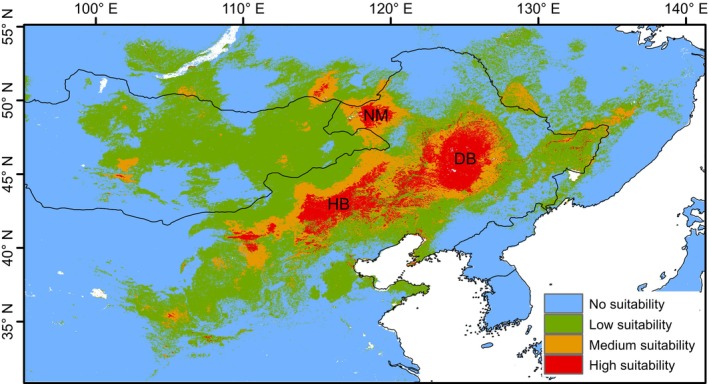
Predicted distribution of the *S. dauricus* .

The results of the SDMs analysis revealed that *S. dauricus* had three main high‐suitability distribution areas, which were consistent with the three populations we hypothesized (Figure 6). Specifically, the largest area is the Northeast Plain, corresponding to the distribution area of the DB population; the second is the Bashang Plateau on the northwest side of the Yanshan Mountains, which is the distribution area of the HB population; and the smallest area is the Hulunbuir Plateau and its adjacent areas with Mongolia and Russia, that is, the distribution area of the NM population (Figure 6). The three high‐suitability distribution areas show fragmented connectivity (e.g., between DB and HB) or no connectivity (e.g., between NM and HB; between DB and NM).

## 4 Discussion

### 4.1 Species Distribution Modeling of *S. dauricus*

The SDM analysis successfully mapped the potential distribution range of *S. dauricus* . The three high‐suitability distribution areas correspond exactly to the three populations of *S. dauricus* identified in this study, namely, the Hulunbuir Plateau population (i.e., the NM population defined herein), the Northeast Plain population (i.e., the DB population defined herein), and the Bashang Plateau population (i.e., the HB population defined herein). *S. dauricus* exhibits high habitat diversity, and habitats including slopes, forest edges, meadows, and fixed sand dunes can all serve as its dwelling places (Fei et al. 1975; Tianbiao et al. 2000). Additionally, *S. dauricus* shows a preference for specific vegetation types, favoring habitats dominated by low‐growing plants of the Gramineae, Asteraceae, and Fabaceae families (Wang, Ji, et al. 2015). In this study, land cover type was also identified as the most important environmental variable in the current distribution model of *S. dauricus* (Table S7). The species response curves revealed that its distribution prefers natural grasslands, wetland grasslands, and riparian grasslands (Figure 5). In addition to land cover, the main environmental variables influencing the distribution model of *S. dauricus* include Annual Mean Temperature, Elevation, and Precipitation Seasonality (Figure 4). The influence of temperature on activity forms the basis of the spatial patterns in annual survival rates of ectotherms. Annual Mean Temperature has a significant negative effect on the hibernation duration and annual survival rate of adult individuals in hibernating species (Turbill et al. 2016). *S. dauricus* is a typical hibernating animal that is dependent on the Annual Mean Temperature. Our species response curves show that it prefers an Annual Mean Temperature range of 0.56°C–7.2°C, with a peak at 3.9°C. Elevation is a critical environmental factor that shapes species distributions, exerting multidimensional influences on species ranges through a series of interconnected physical, climatic, and ecological changes along elevational gradients. Species response curves revealed that *S. dauricus* prefers low‐elevation habitats (< 709 m), with its distribution probability decreasing as elevation increases (Figure 5). It is theorized that more rainfall during the rainy season can lead to higher primary productivity, which in turn increases the reproductive success of some rodent species that feed on seeds and herbs, such as *Akodon olivaceu*, *Octodon degus* , and *Phyllotis darwini* , and results in higher species density (Jaksic et al. 1997; Lima et al. 1999; Madsen and Shine 2009). In this study, the species response curves revealed that greater Precipitation Seasonality is more conducive to the distribution of *S. dauricus* . Specifically, greater Precipitation Seasonality corresponds to greater rainfall in the rainy season, triggering vigorous vegetation growth. Since *S. dauricus* gives birth during the rainy season, the abundant food resources facilitate the breeding and rearing of its offspring.

### 4.2 Population Genetic Relationship of *S. dauricus*

Compared with that of other rodent species (Chiang et al. 2021; Kirkland and Farré 2021; Liu et al. 2019), the nucleotide diversity of the mitochondrial genome of *S. dauricus* in this study was a normal level (Table 1). Among the different populations, the DB population, which had the largest distribution range, presented the highest nucleotide diversity, followed by the HB population. The NM population presented the lowest nucleotide diversity; however, this lower diversity in the NM population may also be related to the small number of sampling sites.

*F*st analysis revealed genetic differences among the three populations (Table S6). In particular, the NM population presented significant genetic divergence from the other two populations, whereas the genetic difference between the DB population and the HB population was the smallest. This pattern was also clearly demonstrated in the PCoA (Figure 3). Additionally, SDM analysis revealed that the distribution area of the NM population is clearly isolated from those of the other two populations, whereas the DB population and the HB population have a fragmented connection. These findings suggest that there is a viable genetic exchange corridor between the DB population and the HB population, whereas potential geographical and genetic barriers may exist between the NM population and the other populations.

AMOVA revealed that although there was considerable genetic variation among the three populations of *S. dauricus* , a greater proportion of the genetic variation originated from differences within individuals (Table S5). Phylogenetic network analysis revealed that although the NM population had a distinct and divergent single clade, this clade still belonged to one of the more variable clades within the DB population and did not exhibit significant differentiation. In addition, phylogenetic network analysis also indicated that although the HB population formed a tight network structure, it was closely embedded within the network of the DB population (Figure 3). The phylogenetic network analysis revealed that the NM and HB populations were embedded within the network structure of the DB population, suggesting that the NM and HB populations originated from the DB population. Therefore, on the basis of the combined results of genetic diversity analysis and genetic structure analysis, we conclude that although genetic differences exist among different populations of *S. dauricus* , they have not yet reached the subspecies level and thus do not support previous classifications of internal subspecies within this species (Wang 2003; Wei 2022).

### 4.3 Demographic History and Human Activity

To respond to cyclical climatic changes in the Pleistocene, species could repetitively expand their ranges via an “expansion‐contraction” strategy during alternating glacial and interglacial periods (Provan and Bennett 2008). All taxa experienced a population decline from the Last Interglacial period to the Last Glacial Maximum (LGM) (Li et al. 2025). Consistently, the BSP analysis in this study revealed that the total population of *S. dauricus* experienced a population contraction before the LGM, followed by a slight expansion of the total population after the LGM (Figure 4). Surprisingly, in the individual BSP analyses of the three populations, only the HB population slightly decreased before the LGM and subsequently expanded after the LGM, whereas the DB and NM populations exhibited no dynamic population changes around the LGM. We hypothesize that this pattern arises because *S. dauricus* is a hibernating species with strong adaptability to low‐temperature climates. Therefore, the climate cooling during the Last Glacial Maximum (LGM) did not exert a drastic impact on the population of the Daurian Ground Squirrel ( *Spermophilus dauricus* ), a species with hibernation habits. Previous studies have shown that in the Hetao Plain near the Bashang Grassland, the vegetation type shifted from a Chenopodiaceae‐Artemisia desert steppe to an Artemisia steppe between 44,000 and 34,000 years ago, which was suitable for the survival of herbivores (Cai et al. 2019). Thus, we propose that the population expansion of the HB population during the same period is associated with the change in vegetation type driven by climate variation in this region.

Consistently, both the BSP analyses of the total population and each of the three individual populations revealed that *S. dauricus* has experienced a severe population decline in the recent period (~5000–3000 years ago to the present). Agriculture emerged in northern China as early as approximately 8000 years ago (Dong et al. 2017; Jia et al. 2024; Lu 2017), whereas grazing appeared around 5500 years ago (Huang et al. 2021). The rapid growth of the human population was bound to lead to conflicts with native wildlife. In particular, *S. dauricus* , a species that primarily feeds on grasses and seeds and thus competes with human interests, has long been regarded as a pest by both herders and farmers. Furthermore, although short‐term and small‐scale overgrazing may increase the population density of *S. dauricus* (Liu et al. 2024), the human utilization intensity of grasslands has undoubtedly increased over thousands of years because of the growth of herder populations and the development of pastoralism. This trend inevitably compresses the food sources of herbivores in the same habitat, resulting in a decline in herbivore populations. This is likely the main reason for the population reduction of the NM population over the past few thousand years. In addition to the impacts of grazing, agricultural development has also encroached on the living space of *S. dauricus* . The SDMs in this study indicate that *S. dauricus* can inhabit farmland habitats excluding rice paddies and greenhouses, possibly because rice‐growing areas are unfavorable for their burrowing behavior. However, rice was domesticated approximately 11,000 years ago (Zhang et al. 2024) and spread to Northeast China approximately 3000 years ago (Liu 2018; Zhu 1996). This may explain the primary cause of the population decline in the DB population over the past millennium. The HB population is located at the intersection of historical nomadic and agricultural development, where both civilizations likely jointly contributed to the reduction of *S. dauricus* populations in the region.

## 5 Conclusion

With the development of human society and population expansion over the past few thousand years, humanity's demands on nature have grown increasingly heavy, making conflicts between humans and nature inevitable. *S. dauricus* is facing such a situation: humans perceive them as causing damage to human life but fail to recognize that these rodents are also confronting the predicament of anthropogenic habitat fragmentation and even habitat loss. The results of this study indicate that all *S. dauricus* populations have experienced a continuous and rapid decline, a phenomenon most likely caused by human production activities. In addition, the NM population, having the lowest nucleotide diversity, should be prioritized for conservation. How we can achieve peaceful coexistence between humans and wild animals is a question worthy of in‐depth reflection.

## Author Contributions

**Xi Chen:** data curation (equal), investigation (equal), software (equal), writing – original draft (equal). **Zhenshan Liu:** data curation (equal), investigation (equal), software (equal). **Ming Yang:** funding acquisition (equal), project administration (equal), writing – review and editing (equal). **Yu Zhou:** conceptualization (equal), methodology (equal), writing – original draft (equal), writing – review and editing (equal).

## Funding

This work was supported by the National Natural Science Foundation of China, 32071518, 32571771, Liaoning Provincial Basic Scientific Research Funds, LJ202410166036, Postgraduate Research and Innovation Program of Shenyang Normal University, YJSCX20250043.

## Conflicts of Interest

The authors declare no conflicts of interest.

## Supporting information


**Table S1:** Information for the *S. dauricus* included in this study.
**Table S2:** Primer information used for mitogenomic Long‐PCR of S. dauricus.
**Table S3:** Site information used for SDM analysis.
**Table S4:** Percentage contribution and permutation importance of all collected environmental variables in Maxent modeling.
**Table S5:** Proportions of total genetic variation within and among the three populations of S. dauricus according to AMOVA.
**Table S6:** Population pairwise FSTs (lower triangle) and FST P values (upper triangle).
**Table S7:** Percentage contribution and permutation importance of environmental variables to a suitable distribution of S. dauricus by the Maxent model.
**Figure S1:** Pearson correlation matrix of 12 high contribution environmental variables.
**Figure S2:** AICc value generated by different combinations of Maxent parameters.
**Figure S3:** The mismatch distribution test of the three populations.
**Figure S4:** ROC curve and AUC value of S. dauricus (10 replicated runs).
**Figure S5:** Relative predictive power of different environmental variables on the basis of the jackknife of regularized training gain in Maxent models.

## Data Availability

The mitochondrial genomes have been deposited in the National Centre for Biotechnology Information (NCBI) Nucleotide with accession numbers PX251470–PX251542 and can be downloaded via the link https://www.ncbi.nlm.nih.gov/nuccore/. The Illumina reads are available in the Sequence Read Archive (SRA) of NCBI under accession numbers SRR35199706–SRR35199778, and can be downloaded via the link https://www.ncbi.nlm.nih.gov/sra/.
