# Supplementary material for: Mitogenomic Relationships and Demographic History of the Daurian Ground Squirrel (Spermophilus dauricus) in Response to Human Activity
Source: Ecol Evol. 2025 Nov 28;15(12):e72605. doi: 10.1002/ece3.72605 (PMC12661366; doi:10.1002/ece3.72605)
Supplement: Supplementary file 1 — Table S1: Information for the S. dauricus included in this study. Table S2: Primer information used for mitogenomic Long‐PCR of S. dauricus. Table S3: Site information used for SDM analysis. Table S4: Percentage contribution and permutation importance of all collected environmental variables in Maxent modeling. Table S5: Proportions of total genetic variation within and among the three populations of S. dauricus according to AMOVA. Table S6: Population pairwise FSTs (lower triangle) and FST P values (upper triangle). Table S7: Percentage contribution and permutation importance of environmental variables to a suitable distribution of S. dauricus by the Maxent model. Figure S1: Pearson correlation matrix of 12 high contribution environmental variables. Figure S2: AICc value generated by different combinations of Maxent parameters. Figure S3: The mismatch distribution test of the three populations. Figure S4: ROC curve and AUC value of S. dauricus (10 replicated runs). Figure S5: Relative predictive power of different environmental variables on the basis of the jackknife of regularized training gain in Maxent models. [file ECE3-15-e72605-s001.docx]

**Supplementary**

Table S1 Information for the *S. dauricus* included in this study.

| Locality code | Voucher | Long | Lat | pop | Coverage depth | GBN |
| --- | --- | --- | --- | --- | --- | --- |
| AES | AES002 | 119.44326 | 47.8095 | NM | 5267 | PX251470 |
| AES | AES003 | 119.44326 | 47.8095 | NM | 5124 | PX251471 |
| EE | EE001 | 119.54838 | 50.21693 | NM | 41 | PX251472 |
| EE | EE002 | 119.54838 | 50.21693 | NM | 4510 | PX251473 |
| EE | EE003 | 119.54838 | 50.21693 | NM | 6801 | PX251474 |
| EE | EE004 | 119.54838 | 50.21693 | NM | 7213 | PX251475 |
| EE | EE006 | 119.54838 | 50.21693 | NM | 7541 | PX251476 |
| EE | EE007 | 119.54838 | 50.21693 | NM | 5701 | PX251477 |
| EE | EE008 | 119.54838 | 50.21693 | NM | 5052 | PX251478 |
| EE | EE009 | 119.54838 | 50.21693 | NM | 4397 | PX251479 |
| EE | EE010 | 119.54838 | 50.21693 | NM | 7705 | PX251480 |
| EE | EE011 | 119.54838 | 50.21693 | NM | 7906 | PX251481 |
| DQ | DQ001 | 124.10774 | 46.67746 | DB | 1740 | PX251482 |
| DQ | DQ002 | 124.10774 | 46.67746 | DB | 7923 | PX251483 |
| DQ | DQ003 | 124.10774 | 46.67746 | DB | 3954 | PX251484 |
| DQ | DQ005 | 124.10774 | 46.67746 | DB | 7425 | PX251485 |
| DQ | DQ006 | 124.10774 | 46.67746 | DB | 6586 | PX251486 |
| DQ | QQ001 | 123.86672 | 46.77926 | DB | 4182 | PX251487 |
| HEB | HEB001 | 126.70408 | 45.87715 | DB | 7061 | PX251488 |
| HEB | HEB002 | 126.70408 | 45.87715 | DB | 727 | PX251489 |
| HEB | HEB003 | 126.70408 | 45.87715 | DB | 6465 | PX251490 |
| HEB | HEB004 | 126.70408 | 45.87715 | DB | 7920 | PX251491 |
| HEB | HEB006 | 126.70408 | 45.87715 | DB | 4911 | PX251492 |
| BC | BC002 | 122.48809 | 12.06268 | DB | 7418 | PX251493 |
| BC | BC003 | 122.48809 | 12.06268 | DB | 7533 | PX251494 |
| SY | SY001 | 123.46865 | 44.17959 | DB | 7889 | PX251495 |
| FK | FK001 | 123.30056 | 42.16323 | DB | 7321 | PX251496 |
| FK | FK002 | 123.30056 | 42.16323 | DB | 5337 | PX251497 |
| FK | FK003 | 123.30056 | 42.16323 | DB | 713 | PX251498 |
| FK | FK004 | 123.30056 | 42.16323 | DB | 527 | PX251499 |
| FK | FK005 | 123.30056 | 42.16323 | DB | 7378 | PX251500 |
| KP | KP002 | 123.26325 | 42.77108 | DB | 4221 | PX251501 |
| KP | KP003 | 123.26325 | 42.77108 | DB | 1039 | PX251502 |
| KP | KP004 | 123.26325 | 42.77108 | DB | 651 | PX251503 |
| KP | KP005 | 123.26325 | 42.77108 | DB | 7693 | PX251504 |
| KP | KP006 | 123.26325 | 42.77108 | DB | 5783 | PX251505 |
| JZ | JZ001 | 121.11992 | 40.87936 | DB | 687 | PX251506 |
| JZ | JZ002 | 121.11992 | 40.87936 | DB | 4995 | PX251507 |
| JZ | JZ003 | 121.11992 | 40.87936 | DB | 2606 | PX251508 |
| JZ | JZ004 | 121.11992 | 40.87936 | DB | 2874 | PX251509 |
| JZ | JZ005 | 121.11992 | 40.87936 | DB | 7638 | PX251510 |
| JZ | JZ006 | 121.11992 | 40.87936 | DB | 5758 | PX251511 |
| JZ | JZ008 | 121.11992 | 40.87936 | DB | 1740 | PX251512 |
| JZ | JZ010 | 121.11992 | 40.87936 | DB | 3418 | PX251513 |
| JZ | JZ013 | 121.11992 | 40.87936 | DB | 7928 | PX251514 |
| JZ | JZ014 | 121.11992 | 40.87936 | DB | 5813 | PX251515 |
| JZ | JZ015 | 121.11992 | 40.87936 | DB | 7924 | PX251516 |
| CF | CF001 | 116.99673 | 42.5387 | HB | 7924 | PX251517 |
| CF | CF002 | 116.99673 | 42.5387 | HB | 5247 | PX251518 |
| XL | XL001 | 116.82755 | 42.31944 | HB | 2138 | PX251519 |
| CD | CD001 | 116.07503 | 41.59463 | HB | 675 | PX251520 |
| CD | CD002 | 116.07503 | 41.59463 | HB | 1325 | PX251521 |
| CD | CD003 | 116.07503 | 41.59463 | HB | 968 | PX251522 |
| CD | CD004 | 116.07503 | 41.59463 | HB | 7969 | PX251523 |
| CD | CD006 | 116.07503 | 41.59463 | HB | 7799 | PX251524 |
| CD | CD007 | 116.07503 | 41.59463 | HB | 5146 | PX251525 |
| CD | CD008 | 116.07503 | 41.59463 | HB | 6907 | PX251526 |
| ZB | ZB001 | 114.74765 | 41.29196 | HB | 7492 | PX251527 |
| ZB | ZB002 | 114.74765 | 41.29196 | HB | 2354 | PX251528 |
| ZB | ZB003 | 114.74765 | 41.29196 | HB | 4715 | PX251529 |
| ZB | ZB004 | 114.74765 | 41.29196 | HB | 4336 | PX251530 |
| ZB | ZB005 | 114.74765 | 41.29196 | HB | 7924 | PX251531 |
| ZB | ZB007 | 114.74765 | 41.29196 | HB | 5247 | PX251532 |
| ZB | ZB008 | 114.74765 | 41.29196 | HB | 1124 | PX251533 |
| DT | DT001 | 113.58566 | 40.35597 | HB | 7899 | PX251534 |
| WL | WL001 | 112.59453 | 41.08083 | HB | 7916 | PX251535 |
| WL | WL002 | 112.59453 | 41.08083 | HB | 2152 | PX251536 |
| WL | WL003 | 112.59453 | 41.08083 | HB | 5675 | PX251537 |
| WL | WL004 | 112.59453 | 41.08083 | HB | 7214 | PX251538 |
| WL | WL005 | 112.59453 | 41.08083 | HB | 7898 | PX251539 |
| WL | WL006 | 112.59453 | 41.08083 | HB | 7921 | PX251540 |
| WL | WL007 | 112.59453 | 41.08083 | HB | 7905 | PX251541 |
| WL | WL008 | 112.59453 | 41.08083 | HB | 5664 | PX251542 |

Table S2. Primer information used for mitogenomic Long-PCR of *S. dauricus.*

| Primer pairs | Primer name | Sequence (5'-3') | Primer positions refer KP708706 |
| --- | --- | --- | --- |
| pair1 | DWE-F4 | TCGTGCCAGCCACCGCGGTCATAC | 316-339 |
|  | DWE-R2 | AAGCTGCGGCTTCAAAGCCGAAGTGATG | 9355-9336 |
| pair2 | DWE-L1 | ATGACCCACCAAACACATGCATACCATATAGT | 8646-8676 |
|  | DWE-R1 | AAGCACCGCCAAGTCCTTTGAGTTTTA | 614-588 |

Table S3 Site information used for SDMs analysis.

| Order | Longitude | Latitude | Source | iNaturalist IDs | Fitness indices |
| --- | --- | --- | --- | --- | --- |
| 1 | 123.86672 | 46.77926 | this study | NA | 0.864091 |
| 2 | 124.10774 | 46.67746 | this study | NA | 0.779385 |
| 3 | 126.70408 | 45.87715 | this study | NA | 0.712866 |
| 4 | 123.46865 | 44.17959 | this study | NA | 0.512452 |
| 5 | 123.26325 | 42.77108 | this study | NA | 0.633924 |
| 6 | 123.30056 | 42.16323 | this study | NA | 0.164328 |
| 7 | 122.48809 | 45.06268 | this study | NA | 0.675716 |
| 8 | 121.11992 | 40.87936 | this study | NA | 0.646105 |
| 9 | 116.07503 | 41.59463 | this study | NA | 0.687311 |
| 10 | 116.99673 | 42.5387 | this study | NA | 0.665419 |
| 11 | 113.58566 | 40.35597 | this study | NA | 0.382885 |
| 12 | 112.59453 | 41.08083 | this study | NA | 0.382846 |
| 13 | 116.82755 | 42.31944 | this study | NA | 0.5956 |
| 14 | 114.74765 | 41.29196 | this study | NA | 0.229826 |
| 15 | 119.44326 | 47.8095 | this study | NA | 0.396679 |
| 16 | 119.54838 | 50.21693 | this study | NA | 0.396314 |
| 17 | 115.7872617 | 43.00565278 | iNaturalist | 212503341 | 0.54447 |
| 18 | 122.0497694 | 41.17053889 | iNaturalist | 7914044 | 0.443085 |
| 19 | 119.7941581 | 49.28904102 | iNaturalist | 287441961 | 0.519492 |
| 20 | 117.6930861 | 48.98456343 | iNaturalist | 182031747 | 0.466932 |
| 21 | 117.1839054 | 43.25166452 | iNaturalist | 142789964 | 0.678981 |
| 22 | 117.5173526 | 43.30259826 | iNaturalist | 152514491 | 0.642443 |
| 23 | 119.765607 | 49.21160714 | iNaturalist | 99413617 | 0.613355 |
| 24 | 114.777025 | 41.36099 | iNaturalist | 87823561 | 0.295155 |
| 25 | 114.8646567 | 41.40734833 | iNaturalist | 87823552 | 0.283391 |
| 26 | 123.4903245 | 41.75267486 | iNaturalist | 46036 | 0.448165 |
| 27 | 117.2544466 | 42.31197833 | iNaturalist | 197503507 | 0.562151 |
| 28 | 124.2775779 | 43.16433464 | iNaturalist | 180766986 | 0.239633 |
| 29 | 119.0517417 | 43.12454167 | iNaturalist | 178329055 | 0.644864 |
| 30 | 119.757669 | 49.50097661 | iNaturalist | 173710240 | 0.542566 |
| 31 | 124.6985302 | 46.671255 | iNaturalist | 216771537 | 0.845936 |
| 32 | 122.3045627 | 45.11722783 | iNaturalist | 213318302 | 0.723838 |
| 33 | 118.613963 | 47.62688634 | iNaturalist | 211531888 | 0.356543 |
| 34 | 117.1586 | 42.55076667 | iNaturalist | 305155639 | 0.662441 |
| 35 | 119.2597815 | 49.31965981 | iNaturalist | 302573602 | 0.895666 |
| 36 | 120.7120499 | 45.41986905 | iNaturalist | 302246735 | 0.432645 |
| 37 | 123.3629198 | 43.50789904 | iNaturalist | 297829962 | NA |
| 38 | 122.4011529 | 45.43389115 | iNaturalist | 287158993 | 0.319995 |

Table S4 Percentage contribution and permutation importance of all collected environmental variables in Maxent modeling.

| Variable abbreviations | Variable Type | Percent contribution | Permutation importance |
| --- | --- | --- | --- |
| LC | Land Cover | 20.2 | 4.1 |
| BIO4 | Temperature Seasonality (standard deviation ×100) | 13.3 | 8.9 |
| BIO13 | Precipitation of Wettest Month | 11.7 | 5.7 |
| BIO1 | Annual Mean Temperature | 10 | 4.6 |
| BIO2 | Mean Diurnal Range (Mean of monthly (max temp - min temp)) | 7.4 | 10.3 |
| BIO19 | Precipitation of Coldest Quarter | 7.2 | 5.5 |
| NDVI | Normalized Difference Vegetation Index | 5.3 | 4.1 |
| ELEV | Elevation | 4.7 | 10.2 |
| BIO17 | Precipitation of Driest Quarter | 4.4 | 5.4 |
| BIO12 | Annual Precipitation | 4.3 | 17 |
| BIO15 | Precipitation Seasonality (Coefficient of Variation) | 4.3 | 1.5 |
| BIO3 | Isothermality (BIO2/BIO7) (×100) | 1.9 | 5.4 |
| BIO14 | Precipitation of Wettest Month | 1.8 | 0.4 |
| BIO8 | Mean Temperature of Wettest Quarter | 1.6 | 16.2 |
| BIO10 | Mean Temperature of Warmest Quarter | 1 | 0 |
| BIO5 | Max Temperature of Warmest Month | 0.6 | 0.4 |
| BIO11 | Mean Temperature of Coldest Quarter | 0.1 | 0 |
| BIO6 | Max Temperature of Warmest Month | 0.1 | 0 |
| BIO18 | Precipitation of Warmest Quarter | 0 | 0 |
| BIO9 | Mean Temperature of Driest Quarter | 0 | 0 |
| BIO7 | Max Temperature of Warmest Month | 0 | 0.3 |
| BIO16 | Precipitation of Wettest Quarter | 0 | 0 |

Table S5 Proportions of total genetic variation within and among the three populations of *S. dauricus* according to AMOVA.

| Source of variation | d.f. | Sum of squares | Variance components | Percentage of variation |
| --- | --- | --- | --- | --- |
| Among populations | 2 | 1091.081 | 22.71591 Va | 39.64 |
| Within populations | 70 | 2421.179 | 35.58827 Vb | 60.36 |
| Total | 72 | 3512.260 | 57.30418 |  |

Table S6 Population pairwise FSTs (lower triangle) and FST P values (upper triangle).

|  | NM | DB | HB |
| --- | --- | --- | --- |
| NM |  | 0.00000 | 0.00000 |
| DB | 0.41192 |  | 0.00000 |
| HB | 0.73030 | 0.21159 |  |

Table S7. Percentage contribution and permutation importance of environmental variables to suitable distribution of *S. dauricus* by Maxent model.

| Variable | | Percent contribution | | Permutation importance | |
| --- | --- | --- | --- | --- | --- |
| LC | | 36.7 | | 5.6 | |
| BIO1 | | 13.5 | | 16.4 | |
| ELEV | | 11.1 | | 6 | |
| BIO15 | | 10.7 | | 2 | |
| BIO13 | | 6.3 | | 26.9 | |
| BIO18 | | 5.5 | | 15.3 | |
| BIO2 | | 5.3 | | 1.5 | |
| BIO3 | | 4 | | 10.9 | |
| NDVI | | 3.1 | | 0.7 | |
| BIO19 | | 1.9 | | 3.3 | |
| BIO4 | | 0.9 | | 2.7 | |
| BIO16 | | 0.8 | | 7.9 | |
| BIO5 | | 0.3 | | 0.9 | |


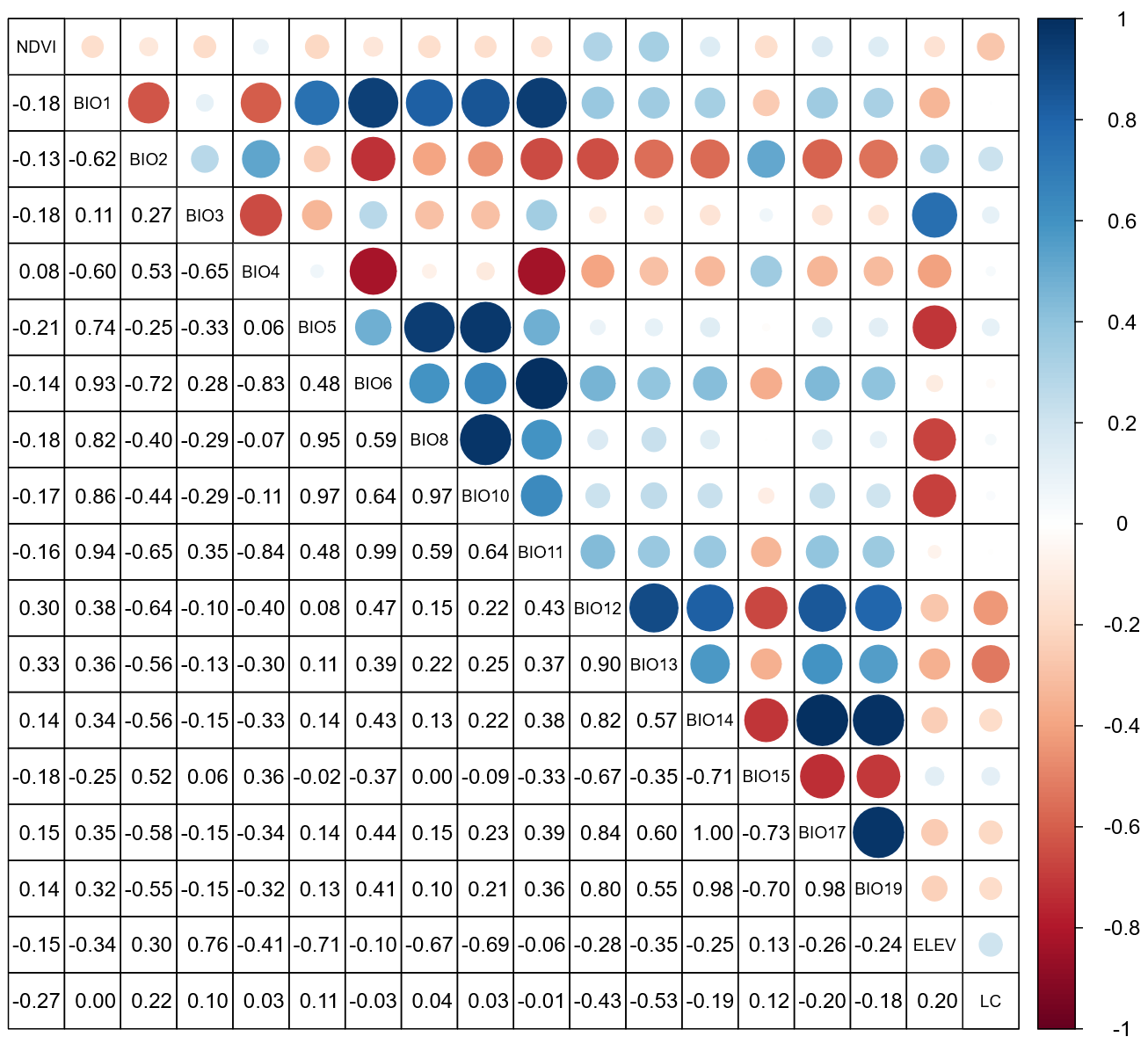


Figure S1 Pearson correlation matrix of 12 high contribution environmental variables.


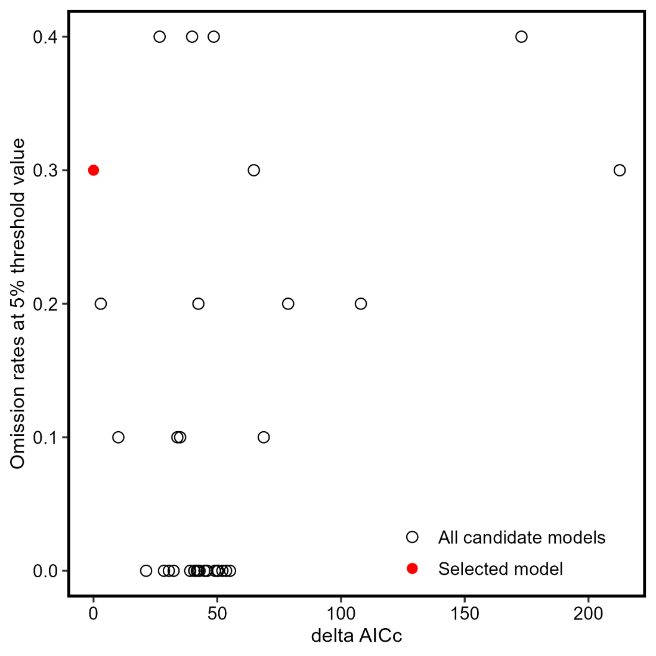


Figure S2 AICc value gengeranted by different combinations of Maxent parameters.


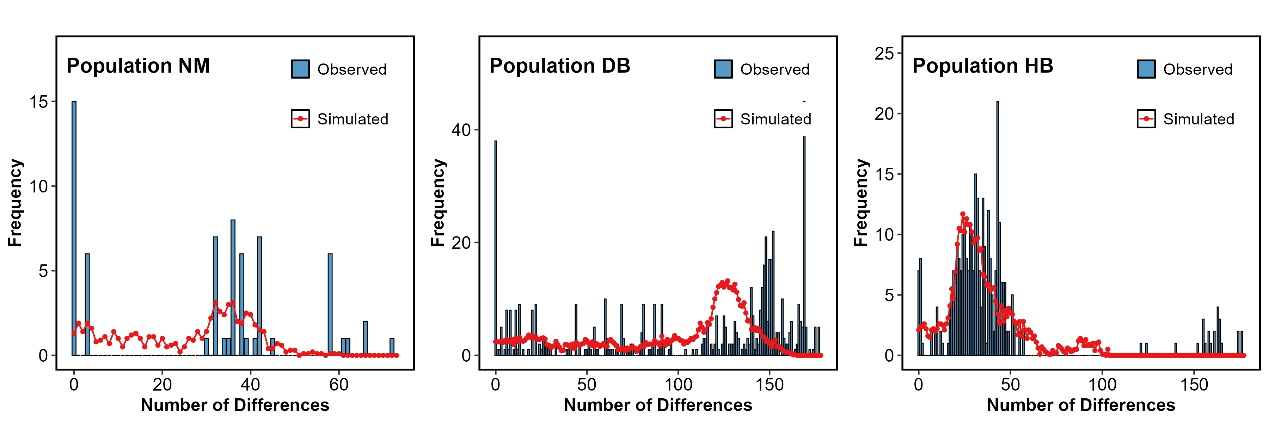


Figure S3 The mismatch distribution test of the three populations.


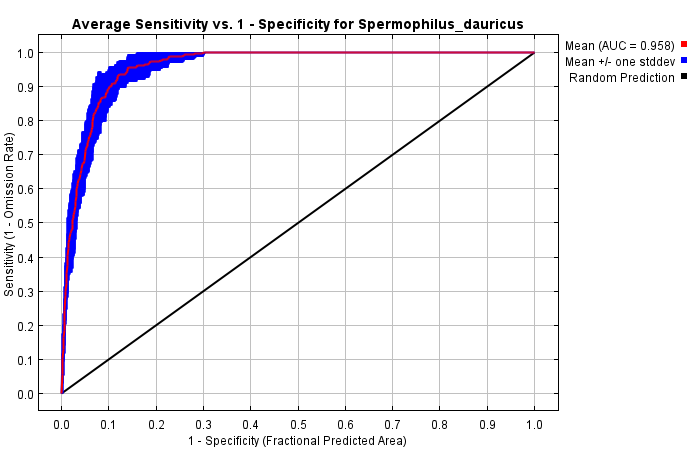


Figure S4 ROC curve and AUC value of *S. dauricus* (10 replicated runs).


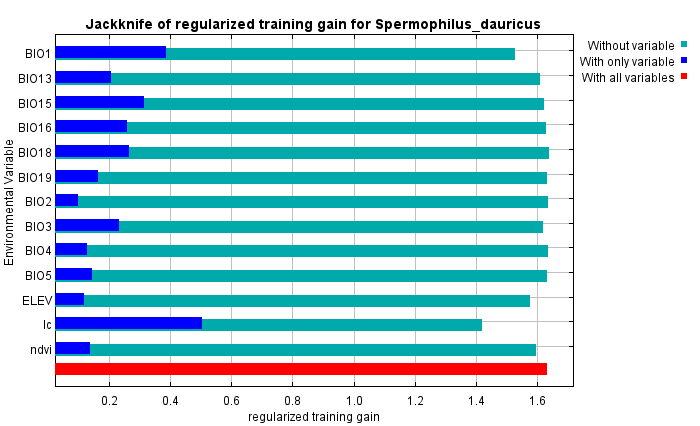


Figure S5 Relative predictive power of different environmental variables based on the jackknife of regularized training gain in Maxent models.
